# Iatrogenic cerebral amyloid angiopathy in older adults

**DOI:** 10.1111/ene.16278

**Published:** 2024-03-21

**Authors:** Larysa Panteleienko, Dermot Mallon, Rupert Oliver, Ahmed Toosy, Yuki Hoshino, Aya Murakami, Kanishk Kaushik, Marieke J. H. Wermer, Hideo Hara, Yusuke Yakushiji, Gargi Banerjee, David J. Werring

**Affiliations:** ^1^ Department of Brain Repair and Rehabilitation, Stroke Research Centre UCL Queen Square Institute of Neurology London UK; ^2^ Department of Neurology Bogomolets National Medical University Kyiv Ukraine; ^3^ National Hospital for Neurology and Neurosurgery, Queen Square University College London Hospitals NHS Foundation Trust London UK; ^4^ Department of Neuroinflammation UCL Queen Square Institute of Neurology London UK; ^5^ Division of Neurology, Department of Internal Medicine Saga University Faculty of Medicine Saga Japan; ^6^ Department of Neurology Kansai Medical University Hirakata Japan; ^7^ Department of Neurology Leiden University Medical Centre Leiden The Netherlands; ^8^ University Medical Centre Groningen Groningen The Netherlands; ^9^ MRC Prion Unit at UCL Institute of Prion Diseases London UK

**Keywords:** amyloid‐β, cadaveric dura mater, iatrogenic cerebral amyloid angiopathy, neurosurgery, prion

## Abstract

**Background and purpose:**

An increasing number of cases of iatrogenic cerebral amyloid angiopathy (CAA) have now been reported worldwide. Proposed diagnostic criteria require a history of medical intervention with potential for amyloid‐β transmission, for example those using cadaveric dura mater or requiring instrumentation of the brain or spinal cord. Clinical presentation occurs after an appropriate latency (usually three or four decades); to date, most patients with iatrogenic CAA have had ‘early‐onset’ disease (compared to sporadic, age‐related, CAA), as a consequence of childhood procedures.

**Results:**

We describe five cases of possible iatrogenic CAA in adults presenting in later life (aged 65 years and older); all had prior neurosurgical interventions and presented after a latency suggestive of iatrogenic disease (range 30–39 years). Use of cadaveric dura mater was confirmed in one case, and highly likely in the remainder.

**Conclusion:**

The presentation of iatrogenic CAA in older adults widens the known potential spectrum of this disease and highlights the difficulties of making the diagnosis in this age group, and particularly in differentiating iatrogenic from sporadic CAA. Increased vigilance for cases presenting at an older age is essential for furthering our understanding of the clinical phenotype and broader implications of iatrogenic CAA.

## INTRODUCTION

Iatrogenic cerebral amyloid angiopathy (CAA), that is, that occurring as a consequence of medical procedures, is now recognized as a distinct form of amyloid‐β CAA. Since the first clinical reports of iatrogenic CAA in 2019 [1], more than 50 cases have been described [2, 3, 4, 5, 6, 7]. Iatrogenic CAA is hypothesized to occur following the transmission of amyloid‐β protein during medical procedures (mainly neurosurgery), with subsequent clinical symptoms of CAA typically developing about three decades after exposure [2, 3]. Nearly all published reports of iatrogenic CAA to date are in patients who had childhood medical procedures, with subsequent symptomatic CAA at an age considered ‘young’ for sporadic CAA (typically under 55 years) [8]. However, there is no biological reason why iatrogenic CAA could not occur at older ages in people who had medical procedures involving exposure to amyloid‐β beyond childhood.

Here, we describe five patients presenting with possible iatrogenic CAA in later life (older than 65 years), defined using recently proposed diagnostic criteria [2].

## CASE REPORTS

**FIGURE 1:**
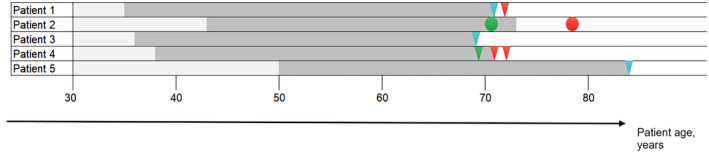
Timeline of the clinical history in patients with iatrogenic CAA. The horizontal axis indicates patient age in years. In the timeline, light grey indicates the period prior to neurosurgery; dark grey indicates the intervals between neurosurgery and CAA diagnosis or first CAA symptoms. Red triangles represent TFNE; red circles represent intracerebral haemorrhage; green triangles represent mild cognitive impairment; green circles represent Alzheimer's disease; blue triangles represent incidental discovery of CAA.

Details of the clinical features and key investigation findings for all cases are summarized in Table 1. The timeline of the clinical presentation is highlighted in Figure 1.

**TABLE 1:** Summary of clinical features and key investigation findings.

		All cases (*n* = 5)	Case 1	Case 2	Case 3	Case 4	Case 5
Age at first presentation, mean (SD), years		73.6 (6.0)	71	73	69	71	84
Sex, male, *n* (%)		3 (60)	F	M	F	M	M
Age at exposure, mean (range), years		40.4 (35–50)	35	43	36	38	50
Latency, mean (range), years		33.8 (30–39)	39	30	33	33	34
Exposure, *n* (%)							
Confirmed cadaveric dura mater use		1 (20)	+	−	−	−	−
Neurosurgery		5 (100)	+	+	+	+	+
Presenting symptom, *n* (%)							
Symptoms not related to CAA		3 (60)	+	−	+	−	+
Cognitive impairment		2 (40)	−	+	−	+	−
ICH		0 (0)	−	−	−	−	−
TFNE		1 (20)	−	−	−	+	−
Associated symptoms, *n* (%)							
Cognitive impairment		2 (40)	−	+	−	+	−
ICH		1 (20)	−	+	−	−	−
TFNE		1 (20)	+	−	−	−	−
MRI biomarkers							
MRI biomarkers of CAA at first MRI							
Lobar CMB	*N* (%)	5 (100)	+	+	+	+	+
cSS	*N* (%)	3 (60)	+	−	+	+	−
Multispot WMH^a^	*N* (%)	1 (20)	−	−	−	+	−
Confluent WMH	*N* (%)	2 (40)	−	+	−	+	−
CSO‐EPVS^b^	*N* (%)	5 (100)	+	+	+	+	+
Progression of MRI biomarkers during follow‐up							
Lobar CMB	*N* (%)	3 (60)	−	+	+	−	+
cSS	*N* (%)	4 (80)	+	−	+	+	+
Multispot WMH^a^	*N* (%)	2 (40)	−	−	−	+	+
Confluent WMH	*N* (%)	3 (60)	−	+	−	+	+

Abbreviations: CAA, cerebral amyloid angiopathy; CMB, cerebral microbleed; cSS, cortical superficial siderosis; CSO‐EPVS, centrum semiovale enlarged perivascular spaces; ICH, intracerebral haemorrhage; MRI, magnetic resonance imaging; TFNE, transient focal neurological episodes (‘amyloid spells’); WMH, white matter hyperintensity.

^a^ Multispot WMH is defined as more than 10 T2‐weighted fluid‐attenuated inversion recovery small circular or ovoid hyperintense lesions in the subcortical white matter of both hemispheres [10].

^b^ Centrum semiovale perivascular spaces, identified on axial T2‐weighted images, are defined as more than 20 visible perivascular spaces in the centrum semiovale of one hemisphere [10].

### Case 1

**FIGURE 2:**
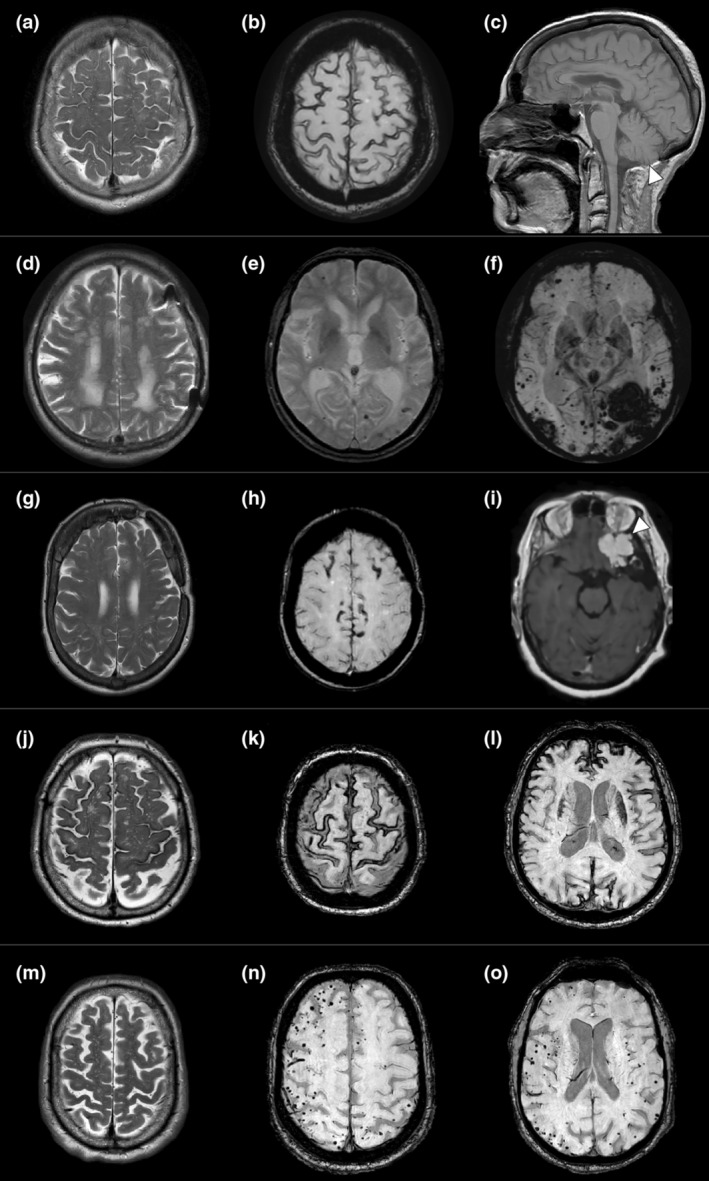
Each row shows representative MRI images for the five patients. The left column shows T2‐weighted imaging. The middle and right column show blood sensitive imaging (either susceptibility‐weighted imaging or gradient‐echo T2‐weighted imaging) unless otherwise stated. Case 1: enlarged perivascular spaces in the centrum semiovale (CSO‐EPVS) (a), cortical superficial siderosis (cSS) (b), and the foramen magnum decompression for a Chiari I malformation ((c) white arrowhead, sagittal T1‐weighted image). Case 2: severe burden of small vessel disease (d) and progressive cerebral microbleeds and the development of an intracerebral haemorrhage (e, f). Case 3: enlarged perivascular spaces (g), convexity cSS (h) and residual sphenoid meningioma ((i) white arrowhead, gadolinium‐enhanced T1‐weighted image). Case 4: enlarged perivascular spaces and multispot pattern of small vessel disease (j) and extensive superficial siderosis (k, l). Case 5: enlarged perivascular spaces (m) and innumerable cerebral microbleeds but no cSS (n, o).

A 74‐year‐old woman presented with a history of episodic severe bilateral headache and episodic forgetfulness for names and facts. There was no history of hypertension. Aged 35 years (1983), she had foramen magnum decompression surgery for a Chiari I malformation (Figure 2c); the operation note confirmed that cadaveric dura mater was used to close a dural defect.

Brain magnetic resonance imaging (MRI) at presentation showed enlarged perivascular spaces in the centrum semiovale (CSO‐EPVS) (Figure 2a), a small number of cerebral microbleeds (CMBs) and cortical superficial siderosis (cSS) (Figure 2b). The headache was controlled with amitriptyline.

One year after presentation, the patient developed transient focal neurological episodes (TFNE), characterized by sensory disturbance (tingling and numbness) involving the right side of the mouth and right hand. Brain MRI performed 1 week later revealed a small convexity subarachnoid haemorrhage over the left central sulcus and some progression of cSS. In the 2 years after this event, she has been clinically and radiologically stable.

### Case 2

A 73‐year‐old man was referred to the memory clinic with a 3‐year history of increased forgetfulness, disorientation and apathy. He had treated hypertension and was an ex‐smoker. Aged 43 years (1987), the patient suffered a traumatic subdural haematoma that was evacuated via a left craniotomy. Cadaveric dura was used routinely for this operation at the treating hospital during this period, but the operative note was unavailable to provide confirmation.

At initial assessment, the patient scored 26/30 on the Mini‐Mental State Examination (MMSE) and 13/18 on the Frontal Assessment Battery. Brain MRI (blood‐sensitive sequences not performed) showed extensive periventricular white matter hyperintensities (Figure 2d). Brain perfusion single photon emission computed tomography (SPECT; using r^99m^Tc‐ECD) revealed hypoperfusion in the posterior cingulate cortex and the precuneus. The patient was diagnosed with Alzheimer's disease.

A repeat brain MRI performed 1 year later showed a high burden of small vessel disease and bilateral exclusively lobar CMBs but no cSS (Figure 2e).

Five years after the original presentation (aged 78 years) the patient was admitted with rapidly progressive cognitive impairment, constructive and orofacial apraxia. RepeatMRI demonstrated an acute left occipital intracerebral haemorrhage (ICH) and many new CMBs (Figure 2f). Cerebrospinal fluid (CSF) analysis demonstrated reduced levels of Aβ‐42 (52.5 pg/mL; normal >620 pg/mL) and reduced Aβ 42/40 ratio (0.072, normal >0.72). The patient's cognitive functions did not improve, and he was transferred to a long‐term care hospital.

### Case 3

A 69‐year‐old woman developed persistent double vision, which was fixed with prism lenses. Aged 36 (1984), she had a subtotal resection of left sphenoid meningioma. Cadaveric dura was used routinely for such operations at the treating hospital at this time, but the operation note was unavailable. The patient developed a persistent (lower) visual field defect following the procedure. There was no postoperative radiotherapy. She suffered from migraines and was not hypertensive. Neuropsychological testing revealed evidence of mild anterior/subcortical dysfunction.

A gadolinium‐enhanced MRI of the brain showed slow growth of the residual meningioma over the previous 14 years (Figure 2i). T2 MRI showed enlarged CSO‐EPVS (Figure 2g); susceptibility‐weighted images (SWI) showed progressive cSS over both cerebral convexities (Figure 2h) and a small number of lobar CMBs (not shown).

### Case 4

A 71‐year‐old man presented with multiple TFNE and increasing forgetfulness over 3 years. These TFNE were characterized by reduced sensation, usually involving left hand, arm and mouth. Neuropsychological testing showed mild cognitive impairment involving verbal memory, executive function and processing speed. The patient was hypertensive and an ex‐smoker. Aged 55 years, he had an acute coronary syndrome requiring coronary artery stenting.

Aged 36 years (1989), the patient had an operation for a herniated disc (L4/L5). The use of cadaveric dura was routine for surgeries requiring durotomy at the treating hospital during this period, but the operative note was unavailable. MRI showed CSO‐EPVS, extensive cSS and lobar CMBs (Figure 2j–l). Over the following 17 months, he continued to experience multiple TFNE, in addition to further cognition deterioration and persisting emotional lability. Follow‐up MRI showed progressive cSS compared to MRI 1.5 years prior.

### Case 5

An 84‐year‐old man was referred with persistent diplopia. After a comprehensive clinical evaluation, the patient was diagnosed with myasthenia gravis and managed with pyridostigmine, with full remission within 5 months. He had a history of hypertension and ischaemic heart disease (coronary artery stenting aged 75 years); cognition was normal. Brain MRI showed CSO‐EPVS and multiple lobar CMBs (Figure 2m–o).

Aged 50 (1986), the patient had an operation for a herniated intervertebral disc. The use of cadaveric dura was routine at the treating hospital during this period, but the operative note was unavailable.

In the following 3 years, repeat brain imaging showed new CMBs and progressive cSS, but the patient remained clinically stable throughout this period.

## DISCUSSION

We describe five cases of possible iatrogenic CAA, all presenting at an age older than previously reported (over 65 years). All patients had prior neurosurgery: one had confirmed and documented cadaveric dura used, whilst the remainder had procedures that were highly likely to have involved cadaveric dural grafting as standard practice in treating hospitals at the time. The latency between the index procedure and clinical presentation in all cases falls within the typical reported range of 30–40 years for iatrogenic CAA. Thus, apart from their older age of onset, these patients fulfil recently proposed diagnostic criteria for iatrogenic CAA regarding exposure, and clinical‐radiological features, at an appropriate latency [2], leading us to conclude that iatrogenic CAA is likely. However, they also meet the age and clinical‐radiological criteria for probable sporadic CAA [9] which is known to be prevalent in this age group. For this reason genetic causes of CAA were not systematically excluded (likely to be less relevant at later ages of presentation) and more invasive tests to demonstrate central nervous system amyloid deposition were not performed (i.e., cerebrospinal fluid, amyloid positron emission tomography, cerebral biopsy), given that amyloid‐β deposition (including asymptomatic deposition) in this age group is common [9].

The clinical presentation of our cases appears to differ from younger‐onset iatrogenic CAA cases reported to date, where ICH (in 83%) and seizures (in 17%) were the most common presenting symptoms [2]. Three cases (1, 3 and 5) were incidentally found to have CAA after investigations for unrelated neurological symptoms (migraine, diplopia) and one of these patients (case 5) remained clinically asymptomatic from their CAA over an extended period of follow‐up (over 3 years). Symptoms typical for CAA (cognitive impairment, TFNE) were observed in the remainder, although it is difficult to comment on their relative frequency compared to previously published cases, given the relatively small number of older cases.

Our cases highlight the challenges of diagnosing iatrogenic CAA in this age group (over 65 years). Genetic testing, which is important in younger cases to exclude differential causes of CAA [8], is less relevant at older ages, where the main alternative diagnosis is sporadic CAA. To date, there are no imaging or molecular biomarkers that allow definitive differentiation between late‐onset iatrogenic versus sporadic CAA. Whilst it is impossible to definitively demonstrate causality, the patients reported here developed CAA at an appropriate latency after a relevant procedure associated with amyloid‐β transmission.

The current diagnostic criteria for iatrogenic CAA were designed in anticipation of older‐onset cases; however, their use in this group to date is limited, allowing only ‘possible’ diagnosis. CAA with young onset is more likely to be reported as it is unusual; it is hoped that our description of suspected iatrogenic CAA at an older age will improve awareness and lead clinicians to consider this diagnosis even in people in the typical age range for sporadic CAA.

## CONCLUSION

This series highlights the widening spectrum of iatrogenic CAA and the importance of asking about prior medical procedures in all CAA patients, regardless of age. Long‐term follow‐up of patients with potential amyloid‐β exposure beyond childhood could help to determine whether CAA occurs more frequently than expected in later life, strengthening the hypothesis that amyloid‐β transmission can occur with exposure beyond childhood. Improved understanding of the clinical phenotype in this older age group and biomarkers for iatrogenic CAA are essential for forming diagnostic criteria.

## AUTHOR CONTRIBUTIONS

**Larysa Panteleienko:** Writing – original draft; data curation; formal analysis. **Dermot Mallon:** Visualization; formal analysis. **Rupert Oliver:** Investigation; data curation. **Ahmed Toosy:** Investigation; data curation. **Yuki Hoshino:** Investigation; data curation. **Aya Murakami:** Investigation; data curation. **Kanishk Kaushik:** Investigation; data curation. **Marieke J. H. Wermer:** Investigation; data curation. **Hideo Hara:** Investigation; data curation. **Yusuke Yakushiji:** Investigation; data curation. **Gargi Banerjee:** Conceptualization; methodology; writing – review and editing; project administration. **David J. Werring:** Conceptualization; methodology; writing – review and editing; project administration.

## CONFLICT OF INTEREST STATEMENT

Nothing to report.

## PATIENT CONSENT FOR PUBLICATION

Case 1 and 3—consent obtained directly from patient; case 2—consent obtained from patient's wife (patient diseased); cases 4 and 5—the patients are part of an ongoing prospective cohort study (the Follow‐up in Sporadic CAA study [FOCAS, NL63256.058.17]) and they consented to have their anonymized data used in international research collaborations concerning CAA.

## Data Availability

The data that support the findings of this study are available on request from the corresponding author. The data are not publicly available due to privacy or ethical restrictions.
